# Deep Learning of GNSS Acquisition

**DOI:** 10.3390/s23031566

**Published:** 2023-02-01

**Authors:** Parisa Borhani-Darian, Haoqing Li, Peng Wu, Pau Closas

**Affiliations:** Department of Electrical and Computer Engineering, Northeastern University, Boston, MA 02115, USA

**Keywords:** GNSS acquisition, machine learning, deep learning, data fusion

## Abstract

Signal acquisition is a crucial step in Global Navigation Satellite System (GNSS) receivers, which is typically solved by maximizing the so-called Cross-Ambiguity Function (CAF) as a hypothesis testing problem. This article proposes to use deep learning models to perform such acquisition, whereby the CAF is fed to a data-driven classifier that outputs binary class posteriors. The class posteriors are used to compute a Bayesian hypothesis test to statistically decide the presence or absence of a GNSS signal. The versatility and computational affordability of the proposed method are addressed by splitting the CAF into smaller overlapping sections, which are fed to a bank of parallel classifiers whose probabilistic results are optimally fused to provide a so-called probability ratio map from which acquisition is decided. Additionally, the article shows how noncoherent integration schemes are enabled through optimal data fusion, with the goal of increasing the resulting classifier accuracy. The article provides simulation results showing that the proposed data-driven method outperforms current CAF maximization strategies, enabling enhanced acquisition at medium-to-high carrier-to-noise density ratios.

## 1. Introduction

The Global Navigation Satellite System (GNSS) [1,2] is the *de facto* technology for position, navigation, and timing (PNT) applications [3,4], when it is available [4,5,6]. The GNSS relies on one or more satellite constellations that transmit ranging signals, which a receiver can use to self-localize. Along the signal processing chain, the first step that is performed by a GNSS receiver is signal acquisition. The outcome of this statistical process decides whether the signal from a particular satellite is present or absent in the received signal, as well as providing a rough estimate of its associated code delay and Doppler frequency, if present. All GNSS receivers [7,8,9] implement such an acquisition process by evaluating the so-called Cross-Ambiguity Function (CAF) and maximizing it [10]. The CAF is a two-dimensional function that is related to the correlation between the received signal and a local code replica for every possible delay/Doppler pair, which is then maximized for signal detection and coarse synchronization. This acquisition process can be regarded as a signal detection problem, where two hypotheses are available: (1) the null hypothesis $H_{0}$ that the signal is not present or not correctly aligned with the local replica; and (2) the alternative hypothesis $H_{1}$ that the signal is present and correctly aligned with the local replica. Three probabilities characterize the performance of the acquisition method: detection (the probability of correctly detecting signal/noise when there is signal/noise); false alarm (the probability of wrongly detecting signal when the satellite is not present); and miss detection (the probability of mistakenly deciding for the null hypothesis when the signal is present). Detection and false-alarm probabilities are used to obtain an important figure of merit in hypothesis tests: the Receiver Operating Characteristic (ROC), which is a plot of the probability of detection as a function of the probability of false alarm [11,12].

Signal acquisition is based on solid statistical grounds, where the approach of maximizing the CAF (i.e., the correlation between the local replica and the incoming signal) can be seen to be optimal under certain model conditions (e.g., Gaussianity of the channel). However, experiments show (e.g., [13]) that reality is typically more challenging and that the assumed nominal model conditions do not always hold true necessarily. Recent attempts to modify the CAF [14,15] to make it more robust to non-Gaussian behaviors (such as heavy-tailed noise distributions) showed outstanding performance, particularly in the context of the GNSS operation under jamming, for instance considering Cauchy [16] or Laplacian [17] distributions, or robust statistics losses [18] in transformed domains [19] or dual domains [20], as well as understanding the impact on positioning solution [21]. Regardless of its remarkable performance in outlier-rich data, the aforementioned robust approach does not accommodate for more complex situations such as multimodal distributions or moderate-to-severe nonlinearities affecting the received signal. In [22], the authors showed preliminary results that such complex behaviors can be learned by employing efficient data-driven methods, trained over large datasets. In particular, the work proposed to use deep neural network (DNN) models to carry out the detection (or classification) process involved in signal acquisition. The current paper extends the methodology presented in [22] to (i) accommodate for enhanced DNN models that improve flexibility and computational complexity through dataset splitting and parallel DNN processing; (ii) allow for noncoherent integration times within the DNN framework through an optimal data fusion step; and (iii) provide a more detailed discussion of results and design tradeoffs for practitioners.

Nowadays, the advent of deep learning as a popular tool has sped up advances in a myriad of disciplines. In short, deep learning algorithms (for instance, the variety of NN architectures currently available) are data-driven models that, instead of using complex-to-derive physics-based models, use large datasets to learn the correlations in the data. This has been recently considered in order to redesign communication receivers using deep learning models [23] with promising results. In the context of the GNSS (boosted by the ever-increasing computational power of receivers [24]), deep learning has been recently investigated in several domains, with [25] providing an excellent summary. Some works explore the use of DNN as multipath mitigation strategies, one of those situations where a physics-based model is either too complex to be used or not available at all. For instance, ref. [26] presented a deep-learning-based beamforming approach to mitigate multipath. That work highlighted the limitations of conventional beamforming algorithms by developing a DNN-based model and applied it in different environments, showing a root mean-squared error (RMSE) reduction. The work in [27] discussed the benefits of DNN in predicting distortions in the urban area, which cause significant degradation to GNSS performance. This is improved by leveraging a DNN to extract useful features from the data to learn GNSS measurement quality for improved prediction (of satellite visibility and pseudorange errors) in urban areas.

The work in [28] proposed an end-to-end deep learning method for satellite selection based on the PointNet and VoxelNet networks as a promising alternative to standard selection procedures. The work in [29] presented a methodology to substitute the CAF calculation (typically performed through local code correlation) by a DNN method that was able to learn the complexities of the multipath channel, with promising results when used in standard tracking loops. Those and other works [30,31] highlight the relevance and popularity that this topic is gaining in the GNSS multipath mitigation challenge [32,33]. On another set of GNSS applications, the impact of the deep learning approaches to counteract GNSS spoofing [34,35,36,37,38] and jamming [39,40] attacks is presented in several works. In the context of the GNSS for Earth sciences, deep learning was considered for earthquake prediction [41], hurricane monitoring [42], ice detection [43], and ionospheric scintillation [44,45,46], as well as in the survey article in [47].

This work investigates the use of Deep Neural Networks (DNNs) for GNSS signal acquisition. In particular, a Convolution Neural Network (CNN) is considered in this paper, used as a binary classifier to determine the presence or absences of signal from a given satellite. The inputs to the CNN are the samples of the CAF, a time/Doppler matrix of correlation values, which can potentially be high-dimensional. The dimensionality prevents its direct use, and thus this paper proposes a divide-and-conquer approach to parallelize the computation employing a bank of CNNs processing smaller CAF portions. The main idea is to split the original image (i.e., the CAF map) into several subimages, such that, for instance, one can operate at the regular sampling frequencies encountered in a GNSS receiver. Each classifier on these subimages produces binary class posteriors that we propose to fuse using a Bayesian rule. The probabilistic results are optimally fused to provide a so-called probability ratio map from which acquisition is decided. The results show remarkable performance, particularly at high signal-to-noise ratio regimes, where the data-driven approach provides enhanced performance when compared with theoretical classification bounds. The reason is related to a more exhaustive use of signal correlation in the neighborhood of the CAF’s peak. This work opens the possibility to further methodological advances in addressing challenging GNSS problems, such as attack detection or other forms of waveform distortions.

The remainder of the paper is organized as follows. Section 2 recalls the basics of standard GNSS signal acquisition. Section 3 details the proposed DNN approach for signal acquisition, including a discussion on the models and its extension to noncoherent integration times. The model training setup is discussed in Section 4, and results are analyzed in Section 5. Finally, conclusions and future research directions are drawn in Section 6.

## 2. GNSS Signal Model and Acquisition

A receiver observes signals from *M* satellites plus noise. After downconversion and sampling (at a rate $f_{s}=1/T_{s}$), the samples’ discrete-time signal is

(1) $$\begin{array}{ccc} y[n] & = & \sum_{i=1}^{M}x_{i}\left[n;\theta_{i}\right]+\eta \left[n\right]\\ x_{i}\left[n;\theta_{i}\right] & = & \alpha_{i}b_{i}\left(nT_{s}-\tau_{i}\right)c_{i}\left(nT_{s}-\tau_{i}\right)e^{j2\pi f_{d}nT_{s}+j\phi_{i}} \end{array}$$

with $\alpha_{i}$ being the amplitude of the *i*-th received signal; $b_{i}\left(\cdot \right)$ the data bits of the *i*-th navigation message; $c_{i}\left(\cdot \right)$ the spreading code of the *i*-th satellite; $\tau_{i}$ the time-evolving delay of the *i*-th satellite; $f_{d,i}$ the Doppler-shift; $\phi_{i}$ a carrier-phase term introduced by the channel; and η[n] models the random noise at the receiver, typically complex, zero-mean, and Gaussian-distributed with variance $\sigma^{2}$. For the sake of clarity, the signal parameters for the *i*-th satellite are gathered in a vector $\theta_{i}={\left(\alpha_{i},\phi_{i},\tau_{i},f_{d,i}\right)}^{⊤}$.

Signal acquisition is one of the first actions a receiver needs to perform, basically deciding whether the signal from a particular satellite is present or absent, as well providing a rough estimation of the code delay and Doppler frequency of the received signal in case it is deemed present [10]. Therefore, when searching for the *i*-th satellite, this problem can be formulated as a hypothesis testing problem with two possibilities:

$$\begin{array}{ccc} H_{0} & : & i-\mathrm{th}\mathrm{satellite}\mathrm{is}\mathrm{not}\mathrm{present}\\ H_{1} & : & i-\mathrm{th}\mathrm{satellite}\mathrm{is}\mathrm{present} \end{array}$$

Equivalently, the two competing hypothesis are

(2) $$\begin{array}{ccc} H_{0} & : & y[n]=\eta [n]\\ H_{1} & : & y\left[n\right]=x_{i}\left[n;\theta_{i}\right]+\eta \left[n\right] \end{array}$$

such that n=0,⋯,N−1 index the *N* samples used in acquisition (i.e., coherent integration interval). Notice that a common approach when deriving GNSS signal acquisition schemes is to omit the inter-satellite interference effects, which are rather low thanks to the quasi-orthogonality of the spread spectrum codes $c_{i}\left(\cdot \right)$. Therefore, the model in (2) only considers the contribution of the *i*-th satellite in (1). Since the parameters in $\theta_{i}$ are unknown, the optimal detection framework (in the maximum likelihood (ML) sense) is the Generalized Likelihood Ratio Test (GLRT), which requires ML estimation (MLE) of the vector $\theta_{i}$. Given a set of *N* observations, $y={\left(y\left[0\right],y\left[1\right],\cdots ,y\left[N-1\right]\right)}^{⊤}$ the MLE of $\theta_{i}$ is defined as

(3) $${\hat{\theta }}_{i}=\arg\max_{\theta_{i}}p\left(y|\theta_{i}\right)\;,$$

where it is typically assumed that the parameters in $\theta_{i}$ are piecewise constant within the *N* samples of y and that the codes have ideal cross-correlation properties, so they can be processed independently at the receiver.

It can be seen that the GLRT results in the maximization of the correlation between the received signal and a locally generated code. This correlation operation is encoded in the so-called Cross-Ambiguity Function (CAF), which is nothing but the correlation between y[n] and the spreading code of the *i*-th satellite at a given delay/Doppler pair (in discrete time):

(4) $$C_{i}\left(\tau ,f_{d}\right)=\frac{1}{N}\sum_{n=0}^{N-1}y\left[n\right]\underset{\mathrm{Local}\mathrm{replica}}{\underset{︸}{c_{i}\left(nT_{s}-\tau \right)\exp\left\{-j2\pi f_{d,i}nT_{s}\right\}}}\;,$$

which can be expressed more compactly in vector notation after gathering *N* samples from the samples and the local code as $y,c_{i}\in C^{N\times 1}$ as

(5) $$C_{i}\left(\tau ,f_{d}\right)=\frac{c_{i}^{H}y}{N}\;.$$

The CAF is crucial in the acquisition (and tracking) of the satellites’ signals. The MLE of $\theta_{i}$ can be expressed in terms of it as

(6) $$\begin{array}{ccc} \left({\hat{\tau }}_{i},{\hat{f}}_{d,i}\right) & = & \arg\max_{\tau ,f_{d}}\left\{{\left|C_{i}\left(\tau ,f_{d}\right)\right|}^{2}\right\} \end{array}$$

(7) $$\begin{array}{ccc} {\hat{\alpha }}_{i} & = & \left|C_{i}\left({\hat{\tau }}_{i},{\hat{f}}_{d,i}\right)\right| \end{array}$$

(8) $$\begin{array}{ccc} {\hat{\phi }}_{i} & = & ∠C_{i}\left({\hat{\tau }}_{i},{\hat{f}}_{d,i}\right)\;, \end{array}$$

and we decide that the *i*-th satellite is present by setting a detection threshold β (designed for a desired false alarm probability) on the test statistic in the optimization problem in (6), such as

(9) $${\left|C_{i}\left(\tau ,f_{d}\right)\right|}^{2}\overset{H_{1}}{\underset{H_{0}}{≷}}\beta \;.$$

### 2.1. CAF Evaluation

The CAF is therefore a function which depends on the delay τ and the Doppler frequency $f_{d}$ of the local replica. The optimization in (6) is performed over a grid of possible τ and $f_{d}$ values, typically evaluating the CAF on a set of discrete values. Such a bidimensional grid is referred to as the search space. The search space consists of a set of cells which include the different value of delay and Doppler, which we gather in vectors $\tau \in R^{n_{\tau }}$ and $f_{d}\in R^{n_{f}}$, respectively. Typically, we have that $n_{\tau }\gg n_{f}$. The evaluation of this grid can be performed following several strategies that trade off search speed and performance. Three searching strategies are typically considered: maximum search, serial search, and hybrid search strategies [10].

1. Maximum: This strategy evaluates the CAF all over the search space $R^{n_{\tau }}\times R^{n_{f}}$, such that each cell corresponds to a CAF value at the corresponding delay/Doppler pair. The overall maximum value of the ambiguity function is then selected and compared with the threshold β, if the maximum’s value is greater than β, the satellite is considered acquired, with the estimated code delay and Doppler frequency corresponding to those of the maximum’s cell.
2. Serial: In this strategy, the ambiguity function is evaluated serially cell by cell. In each cell, when the ambiguity function (9) is computed, it is immediately compared with the threshold. If the value exceeds the threshold, the acquisition process stops, and the value of the estimated code delay and Doppler frequency are matched to those from the cell under the test. This strategy has the benefit of reducing the number of CAF evaluations, at the expense of some performance degradation.
3. Hybrid: This strategy evaluates the ambiguity function row by row (or column by column), and at the end of each row (column), the values of the computed ambiguity functions are compared with the threshold. As soon as the maximum value in the current row (column) exceeds the threshold, the acquisition process stops, and the estimated code delay and Doppler frequency are set to the corresponding cell. This strategy brings in a balance between the two approaches above.

In this work, we consider the maximum search strategy, both for the standard GNSS acquisition revisited in this section and the DNN approach proposed in the upcoming Section 3.

### 2.2. Benchmark Performance Using the Receiver Operating Characteristic Function

The so-called Receiver Operating Characteristic (ROC) is a popular metric to assess the performance of any detector/classifier. An ROC is a plot of the detection probability ($P_{d}$) as a function of the false alarm probability ($P_{fa}$). More precisely, $P_{d}=P\left\{{\left|C_{i}\left(\tau ,f_{d}\right)\right|}^{2}>\beta |H_{1}\right\}$ is the probability of correctly detecting a GNSS signal given that it was present, while $P_{fa}=P\left\{{\left|C_{i}\left(\tau ,f_{d}\right)\right|}^{2}>\beta |H_{0}\right\}$ is the probability of detecting the signal given that it should have not been detected. Ideally, the aim is to have the classifier operate such that $P_{d}\to 1$ and $P_{fa}\to 0$.

Theoretical ROC curves are well known for GNSS signal acquisition [10] and used to benchmark different algorithmic solutions. The remainder of this section provides a quick summary of the theoretical ROC used in coherent/noncoherent integration schemes. In this article, we use this theoretical ROC to assess the performance of our DNN-based solution against the best achievable performance under standard (i.e., non-data-driven) method.

In order to calculate the ROC curves, first, one needs to calculate the $P_{fa}$ and $P_{d}$ probabilities. The value of the detection threshold β is typically computed for a given false-alarm probability, given by

(10) $$P_{fa,K}\left(\beta \right)=\exp\left(-\frac{\beta }{2\sigma_{n}^{2}}\right)\sum_{k=0}^{K-1}\frac{1}{k!}{\left(\frac{\beta }{2\sigma_{n}^{2}}\right)}^{k}$$

where *K* indicates the number of noncoherent integrations (i.e., averages of *K*-coherent integrations, as in (9)) considered (such that K=1 in the absence of noncoherent integration) and $\sigma_{n}^{2}=\frac{\sigma^{2}}{2N}$ is the variance of the in-phase and quadrature outputs.

Then, the $P_{d}$ can be calculated as a function of β as

(11) $$P_{d,K}\left(\beta \right)=Q_{K}\left(\sqrt{K\frac{\lambda }{\sigma_{n}^{2}}},\sqrt{\frac{\beta }{\sigma_{n}^{2}}}\right)$$

where $\lambda =\alpha_{i}^{2}/4$ is the noncentrality parameter, and the generalized Marcum *Q*-function is defined as

(12) $$Q_{K}\left(a,b\right)=\frac{1}{a^{K-1}}\int_{b}^{+\infty }x^{K}\exp\left(-\frac{a^{2}+x^{2}}{2}\right)I_{K-1}\left(ax\right)dx\;,$$

which allows for computation of the ROC curves.

## 3. Deep Learning Method for GNSS Acquisition

**Figure 1:**
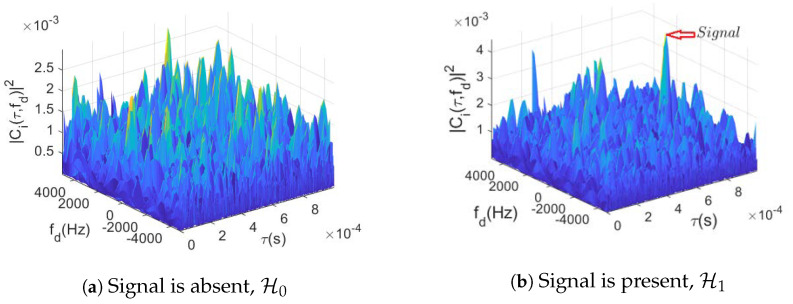
CAF evaluation at the delay/Doppler grid in the (**a**) absence and (**b**) presence of a signal with $C/N_{0}=39$ dB-Hz.

In this work, the goal is to create a neural network model that is capable of recognizing the presence/absence of satellite signals from CAF maps. To that aim, we use as inputs the CAF evaluated at the delay/Doppler grid, which can be regarded as images from the machine learning perspective. Such images (refer to Figure 1 for an exemplary situation) have certain characteristics that can be used to determine whether the signal is present or not, namely: (i) in the absence of signal from a specific satellite, the image should be composed of random values (theory telling that the CAF would be exponentially distributed in that case); and (ii) in the presence of a satellite, a peak should emerge from the random noise floor. This knowledge can be used to train a data-driven model (e.g., a neural network of some sort), such that a classifier can be used which learns to discriminate between $H_{0}$ and $H_{1}$, the hypotheses described earlier in Section 2.

The framework presented in this work is independent of the particular NN architecture, although a Convolution Neural Network (CNN) is used without loss of generality. CNNs are very popular within the computer vision community thanks to their ability to capture complex nonlinear phenomena at the expense of larger complexity compared with multilayer perceptrons (or MLPs). The CNN model is discussed in this section after a brief overview of how the classifier is built following a probabilistic approach. The NN is used in order to provide Bayesian estimates of the hypotheses’ probabilities given the observed data.

### 3.1. Data-Driven, Physics-Based Signal Acquisition

This section formulates the probabilistic hypothesis test, which in this work is solved through a data-driven approach. More precisely, the proposed approach is also informed by the nominal model discussed in Section 2, whereby the CAF for a given satellite $C_{i}\left(\cdot ,\cdot \right)$ is computed in order to extract the signal from the noise floor, thus enabling acquisition by the data-driven model. The intuition is that the *physics* of the problem are accounted for (that is, the optimal solution using the CAF), while augmented with a data-driven model in the vein of [48].

The data fed to the NNs are the CAF’s delay/Doppler map for the *i*-th satellite, which we denote with $Z_{i}\in R^{n_{\tau }\times n_{f}}$ in the sequel. The proposed methodology works on a per-satellite basis. That is, the {m,n} element of the input matrix is defined as

(13) $${\left[Z_{i}\right]}_{m,n}=|C_{i}\left({\left[\tau \right]}_{m},{\left[f_{d}\right]}_{n}\right){|}^{2}\;,$$

where τ and $f_{d}$ are vectors containing the computed delay and Doppler-shifts, respectively. We use the convention that ${\left[a\right]}_{m}$ represents the *m*-th element in the vector, a, and that ${\left[A\right]}_{m,n}$ provides a shortcut for the element of A in the *m*-th row and *n*-th column.

In the Bayesian sense, the information of the models is gathered in their a posteriori distribution after observing the data. An optimal (Bayesian) test between $H_{0}$ and $H_{1}$ is given by the ratio,

(14) $$\frac{p(H_{1}|Z_{i})}{p(H_{0}|Z_{i})}\overset{H_{1}}{\underset{H_{0}}{≷}}1\;,$$

in which case we basically favor the model with the largest a posteriori probability. This can be further expanded in terms of the likelihood and a priori distributions as

(15) $$\frac{p(Z_{i}|H_{1})}{p(Z_{i}|H_{0})}\frac{P(H_{1})}{P(H_{0})}\overset{H_{1}}{\underset{H_{0}}{≷}}1\;,$$

where we readily identify that $P(H_{i})$ denotes the a priori probability of the *i*-th hypothesis. In the absence of better priors, we may assume equally likely hypotheses $P\left(H_{0}\right)=P\left(H_{1}\right)=1/2$. Otherwise, we might incorporate that information in the hypothesis test, resulting in the adjustment of a threshold γ. The resulting test statistic is such that

(16) $$T\left(Z_{i}\right)≜\frac{p(Z_{i}|H_{1})}{p(Z_{i}|H_{0})}\overset{H_{1}}{\underset{H_{0}}{≷}}\gamma \;,$$

which would substitute the standard acquisition test defined in (9). Since the test statistic is a ratio of probabilities, we have that $0<T(Z_{i})<\infty$.

The trained NNs (explained below) are then providing the probabilities of each of the two hypotheses in (16). Therefore, the input data would be $Z_{i}$ and the output of the NN would be the estimated probability for the *i*-th satellite to be absent or present in the dataset y used to build $Z_{i}$.

If the test in (16) results in favor of $H_{1}$, then an estimate of the delay/Doppler for the *i*-th satellite is given by the arguments of the largest element in $Z_{i}$. That is,

(17) $$\left\{\hat{m},\hat{n}\right\}=\arg\max_{m,n}\;T\left({\left[Z_{i}\right]}_{m,n}\right)$$

such that ${\hat{\tau }}_{i}={\left[\tau \right]}_{\hat{m}}$ and ${\hat{f}}_{d,i}={\left[f_{d}\right]}_{\hat{n}}$. As a consequence, once a signal is detected at a specific delay/Doppler bin, that would become the coarse estimate for those parameters.

NNs are models composed of neurons, which are information processing units for complex data processing. An NN consists of an input layer, one or more hidden layers, and an output layer, as well as predefined activation functions that connect adjacent layers. Each layer has a specific weight, which is usually determined with backpropagation during a training process that involves large amounts of data with known labels [49,50]. The network design process is important in order to achieve high accuracy while keeping the network complexity within feasible bounds. Some aspects are effective in designing the network, such as the number of layers, number of neurons, and type of optimizer. In this work, we considered the pretrained VGG16 neural network model as a baseline, where some heuristic modifications to adapt those hyperparameters to the problem at hand were implemented after several trials. The use of VGG16 is common in image processing tasks, which resembles the type of classification challenges the proposed algorithm needs to tackle. More automated approaches to select the NN architecture can be considered in future works, such as the use of Bayesian Optimization [51]. Additional details on the NN structure are provided in Section 4.

### 3.2. Model Structure

**Figure 2:**
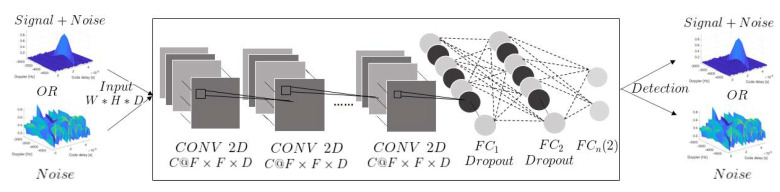
Classification of signal ($H_{1}$) or noise ($H_{0}$) in CAFs as part of the proposed GNSS signal acquisition scheme. Particularly, a set of convolutional layers followed by fully connected layers provide the capabilities of deep learning from large datasets.

CNNs are one of the most popular models for deep learning, with demonstrated performance in label classification in the context of image datasets. A CNN can have tens or hundreds of layers, where each of these layers learn to identify different features of an image [52,53]. At each layer, a cascade of filters is applied to input images, whose parameters were previously learned from pairs of known input/output images. The output of each layer is used as an input to the next layer sequentially. Figure 2 illustrates a CNN structure, as employed in this work. In contrast to other neural networks such as MLPs, CNNs are composed of an input, convolutional layers (whereby the image is filtered through convolution with filters learned from the data), several fully connected hidden layers, and an output layer. During training, the input size of the CNN is fixed; the input goes through a stack of convolutional layers with the same or different filter sizes. In each convolution layer, the filter sweeps the input image from left to right and up to down by using a stride with a size of 2 pixels, which is the number of pixels for each time the filter shifts. In the end, the convolution layers are followed by fully connected (FC) layers and a final *softmax* layer, which is used for classification purposes and produces the desired class probabilities [53].

The CNN structure is shown in the central box of Figure 2, which features several convolution and fully connected layers. Each convolution layer consists of a number of filters (*C*), with filter size (*F*) and channel size (*D*). The *ℓ*-th convolutional layer transforms its input images from the previous layer with dimensions of $W_{\ell -1}\times H_{\ell -1}\times D_{\ell -1}$ through a set of convolution filters, each of these filters activates certain features from the images and creates an output with dimensions of $W_{\ell }\times H_{\ell }\times D_{\ell }$ as an input to the next layer. Notice that initial dimensions are such that $W_{0}=n_{\tau }$ and $H_{0}=n_{f}$, whereas $D_{\ell }=1$, ∀ℓ, since the data are matrices.

After each layer, a batch normalization is used to speed up learning, and an activation function is employed before generating the layer output. The number of convolution layers depends on the structure that is used, where the tradeoff is between complexity (reduced number of convolutional layers) and performance (high accuracy). After the last convolution layer, the CNN architecture has a set of fully connected layers in charge of the classification task, and the output of these last layer has the dimensions of the number of classes (two in the case of this article, where a binary test is solved in (2)) that will be predicted. The output would be the predicted probabilities for each class, as required to compute the test in (16).

**Figure 3:**
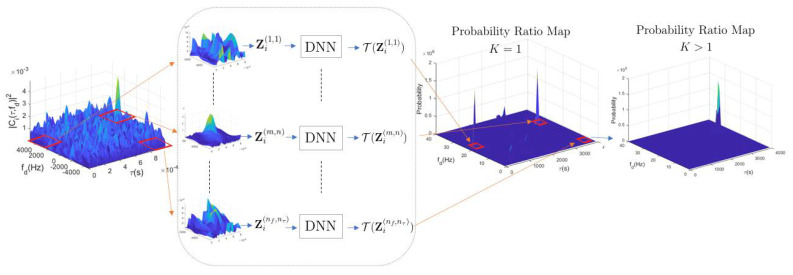
Proposed acquisition method, where the CAF map $Z_{i}$ is split into smaller subimages $Z_{i}^{\left(m,n\right)}$ which are fed to a bank of parallel DNN binary classifiers to produce probability ratio maps. To increase accuracy, several (K>1) probability ratio maps can be noncoherently fused, as shown in the rightmost plot.

The main objectives of this work are to classify the absence/presence of satellite signals in CAF maps, as well as to accurately estimate their delay/Doppler parameters in case of their positive detection. To achieve the latter, a CAF map is computed in a dense delay/Doppler grid—as it is common for standard acquisition schemes—which is then fed to the NN model in charge of producing the posterior class probabilities. As a consequence, the input matrix size can be potentially large (i.e., $n_{\tau }n_{f}$), which might not only pose a computational complexity limit but also increase the expense, since the processing device needs a GPU with larger memory. In order to alleviate this issue, a sliding scheme is proposed in this work, whereby the large-input CAF matrix is scanned using lower dimensional images as the input to the NN classifier. More precisely, the input dataset image is split into several subimages, each corresponding to a test delay/Doppler value. The objective being to reduce the initial dimensions $W_{0}$ and $H_{0}$, such that the processing is computationally affordable and parallelized. These subimages are separately fed to multiple (parallelized) NNs that provide the corresponding class probability conditional on a specific delay/Doppler hypothesis or bin. The concept is sketched in Figure 3, where the {m,n}-th subimage corresponds to the correct location of the delay/Doppler. The output of the DNN structure, labeled as K=1 in the plot, is the probability ratio map derived by the Bayesian hypothesis test.

**Figure 4:**
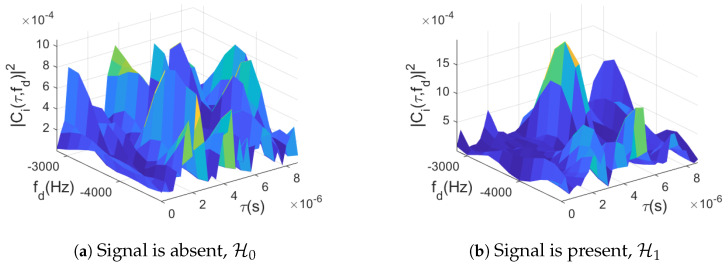
Portions of the CAF fed for processing to the NN with $\Delta_{m}=18$ and $\Delta_{n}=5$ defining the size of the {m,n}-th subimage The resulting subimage $Z_{i}^{\left(m,n\right)}$ is shown on the reduced delay/Doppler grid in the case of the (**a**) absence and (**b**) presence of a GNSS signal with $C/N_{0}=39$ dB-Hz. In the absence of signal, samples are i.i.d., while a time–frequency correlation can be observed in the presence of signal.

The subimages are possibly overlapping regions of the full CAF map, which are centered at a specific delay/Doppler bin hypothesis. While this might increase the overall computational complexity, it enables direct parallelization of the task, which would counteract such complexity. Recall that indices *m* and *n* map to the corresponding delay ${\left[\tau \right]}_{m}$ and Doppler ${\left[f_{d}\right]}_{n}$ values, respectively. Therefore, the {m,n}-th subimage for the *i*-th satellite will be defined as

(18) $$Z_{i}^{\left(m,n\right)}={\left[Z_{i}\right]}_{m+\delta_{m},n+\delta_{n}}$$

where $\delta_{m}=\left[-\Delta_{m},\cdots ,0,\cdots ,\Delta_{m}\right]$ and $\delta_{n}=\left[-\Delta_{n},\cdots ,0,\cdots ,\Delta_{n}\right]$ for some positive integers $\Delta_{m},\Delta_{n}\in Z^{+}$, thus resulting in a su-image dimension of $\left(2\Delta_{m}+1\right)\times \left(2\Delta_{n}+1\right)$, which is much smaller than the original CAF dimension of $n_{\tau }\times n_{f}$. Figure 4 provides an example of an arbitrary subimage $Z_{i}^{\left(m,n\right)}$. As a consequence of the splitting image approach, the statistical test in (14) is in reality implemented for each subimage, such that

(19) $$T\left(Z_{i}^{\left(m,n\right)}\right)≜\frac{p(H_{1}|Z_{i}^{\left(m,n\right)})}{p(H_{0}|Z_{i}^{\left(m,n\right)})}\overset{H_{1}}{\underset{H_{0}}{≷}}1$$

is computed for each {m,n} pair, resulting in a *probability ratio map* (in contrast to the CAF map) for every test delay and Doppler value in τ and $f_{d}$. Recall that $m=\{1,\cdots ,n_{\tau }\}$ and $n=\{1,\cdots ,n_{f}\}$.

It is worth noting that the probability ratio map may contain false peaks, as shown in Figure 3 under K=1. To mitigate those potential false detections, Section 3.3 describes a methodology to fuse noncoherent integrations of *K* DNN outputs. The effect of those integrations is depicted in Figure 3 in the rightmost panel for K=6 noncoherent integrations, where the signal probability is accentuated in the correct delay/Doppler bin, while false peaks arising from noise are attenuated in the fused probability ratio map.

### 3.3. Noncoherent Integration through Fusion of Classifiers

Coherent integration of long code sequences can be implemented in computing the CAF map, $C_{i}\left(\cdot ,\cdot \right)$, in the usual manner. In implementing noncoherent integrations, an alternative is to fuse the multiple probability ratio maps resulting from processing CAF images through the NN architecture described earlier in Section 3.2. We denote by $K\in Z^{+}$ the total number of noncoherent integrations. This section discusses the data fusion of such multiple classifiers. It is known that increasing integration time (both coherently and noncoherently) improves the overall detection performance of the acquisition process, this same rational holds in the case of the data-driven classifier proposed here, whereby noncoherent integrations (i.e., fusion of multiple classifier solutions) improves the reliability of the so-called probability maps (i.e., by attenuating falsely detected peaks or enhancing locations where actual signals reside).

When processing noncoherent snapshots of data, a set of *K* CAF maps is computed. In the standard approach, this would correspond to full CAF maps $Z_{i,k}$ with k=1,⋯,K. In the subimage approach, the result is a different subimage for every integration period, $Z_{i,k}^{\left(m,n\right)}$. In order to combine the class probabilities of the *K* classifiers (which are assumed conditionally independent given their own data), we use Bayes’ rule to derive an optimal fusion rule. For an arbitrary {m,n} pair, the optimal Bayes detector based on the *K* noncoherent integrations is

(20) $$T\left(Z_{i,1}^{\left(m,n\right)},\cdots ,Z_{i,K}^{\left(m,n\right)}\right)=\frac{p(H_{1}|Z_{i,1}^{\left(m,n\right)},\cdots ,Z_{i,K}^{\left(m,n\right)})}{p(H_{0}|Z_{i,1}^{\left(m,n\right)},\cdots ,Z_{i,K}^{\left(m,n\right)})}\overset{H_{1}}{\underset{H_{0}}{≷}}1\;,$$

where, by using the conditional independence assumption of the *K* snapshots, we obtain

(21) $$\begin{array}{c} p\left(H_{1}|Z_{i,1}^{\left(m,n\right)},\cdots ,Z_{i,K}^{\left(m,n\right)}\right)=\frac{P\left(H_{1}\right)\prod_{k=1}^{K}p\left(Z_{i,k}^{\left(m,n\right)}|H_{1}\right)}{p(Z_{i,1}^{\left(m,n\right)},\cdots ,Z_{i,K}^{\left(m,n\right)})}\\ \propto {\left[\prod_{k^{\prime }=1}^{K-1}P\left(H_{1}\right)\right]}^{-1}\prod_{k=1}^{K}p\left(H_{1}|Z_{i,k}^{\left(m,n\right)}\right) \end{array}$$

and

(22) $$\begin{array}{c} p\left(H_{0}|Z_{i,1}^{\left(m,n\right)},\cdots ,Z_{i,K}^{\left(m,n\right)}\right)=\frac{P\left(H_{0}\right)\prod_{k=1}^{K}p\left(Z_{i,k}^{\left(m,n\right)}|H_{0}\right)}{p(Z_{i,1}^{\left(m,n\right)},\cdots ,Z_{i,K}^{\left(m,n\right)})}\\ \propto {\left[\prod_{k^{\prime }=1}^{K-1}P\left(H_{0}\right)\right]}^{-1}\prod_{k=1}^{K}p\left(H_{0}|Z_{i,k}^{\left(m,n\right)}\right) \end{array}$$

which explicitly contain the binary class probabilities of the *K* classifiers: $p(H_{0}|Z_{i,k}^{\left(m,n\right)})$ and $p(H_{1}|Z_{i,k}^{\left(m,n\right)})$. The statistical test can then be formulated as

(23) $$T\left(Z_{i,1}^{\left(m,n\right)},\cdots ,Z_{i,K}^{\left(m,n\right)}\right)=\prod_{k=1}^{K}\frac{p(H_{1}|Z_{i,k}^{\left(m,n\right)})}{p(H_{0}|Z_{i,k}^{\left(m,n\right)})}\overset{H_{1}}{\underset{H_{0}}{≷}}{\left(\frac{P(H_{1})}{P(H_{0})}\right)}^{K-1}≜\gamma$$

such that the decision threshold becomes γ=1 when $P\left(H_{0}\right)=P\left(H_{1}\right)$. It can be observed that the optimal fusion rule is to multiply the *K* binary class probabilities (similar to what was shown in [54]) resulting from the *K* noncoherent integrations processed by the NN classifier. The role of the decision threshold is relevant, as is discussed later, in establishing the $P_{d}$ and $P_{fa}$ of the overall classifier. A reasonable choice is to assume that both hypotheses are equally probable, such that γ=1.

**Figure 5:**
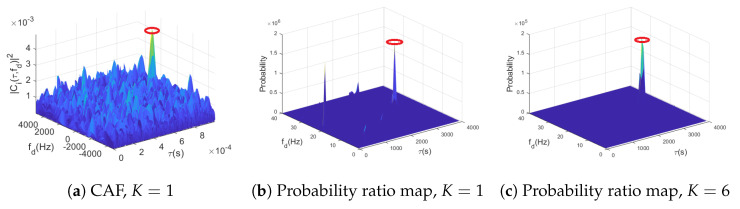
Comparison of the delay/Doppler grid for (**a**) standard CAF map with coherent integration only, (**b**) probability map produced by the data-driven classifier with coherent integration only, and (**c**) probability map after fusing K=6 noncoherent classifier outputs. The GNSS signal had a $C/N_{0}$ of 42 dB-Hz and the red circled highlights the location of the peak generated by the GNSS signal.

A qualitative example of how the fusion rule impacts the performance of the classifier is provided in Figure 5. On the one hand, Figure 5a shows the CAF delay/Doppler map used in standard signal acquisition without any noncoherent integration and just 1 ms coherent integration. It can be seen, as it is known from the GNSS literature, that outside the true peak (denoted with a red circle) the noise floor is relatively spiky and can cause substantial false alarms, particularly at low $C/N_{0}$ values. On the other hand, the proposed data-driven method takes the CAF values and processes them to produce the so-called probability ratio maps, as defined on the right-hand side of (23). The probability ratio map resulting from processing the CAF in Figure 5a can be observed in Figure 5b, where it is clear that the variability in the noise floor was reduced, although residual spikes can still be detected at delay/Doppler bins where no signal was present. This effect is smoothed further with the fusion method, as shown in Figure 5c, where K=6 noncoherent integrations were considered. Notice that the NN uses subimages as inputs to produce a class probability pair, as depicted in Figure 4. As a consequence, the posterior probabilities are taking into consideration the delay/Doppler correlations of the CAF around the signal peak, in contrast to the standard method which only considers the maximum value of the CAF, thus neglecting the waveform arising from the noise form (i.e., the autocorrelation function of the corresponding spreading code).

## 4. Model Training

This section provides details on how the model was trained. Particularly, we used a realistic GNSS signal simulator to generate I&Q samples from GPS L1 C/A satellites with various parameters according to the training plan described here. In order to increase the detection and localization accuracy, a larger sampling frequency might be desirable, since that accentuates the correlated samples around the CAF peak and helps in increasing the accuracy. However, this has an impact on the number of samples to be processed, and a tradeoff needs to be considered. Therefore, here, we increased the sampling frequency to 4 MHz, compared with the 2 MHz that was considered in our preliminary work [22]. As discussed earlier in Section 3.2, increasing the sampling frequency can make the CAF image become high-dimensional if applied directly to a DNN model for classification. That would make the use of DNN more complex and expensive; in that case, the processing device might be required to have a GPU with larger memory to process the GNSS acquisition. In order to reduce the complexity and the expense regardless of increasing $f_{s}$, the full CAF image is split, and a sliding DNN scheme is considered in this work.

More precisely, a dataset consisting of three thousand snapshots of GPS L1 C/A, I&Q samples was generated for model training purposes. The dataset consisted of a range of representative carrier-to-noise-density ratios ($C/N_{0}$) varying between 33 and 45 dB-Hz. The length of these snapshots was 1 ms, the duration of a code, such that this constitutes the coherent integration time of the approach. Additionally, the dataset was generated with random delays between 0 to 1 ms and Doppler shifts between −4000 and 4000 Hz. These I&Q samples were then processed to compute the CAF maps over the Doppler-delay grid, which is then split and processed through the DNN model considered in this article. An analogy to images can be made for these CAFs, where each Doppler/delay cell is a pixel whose value is that of the CAF, $Z_{i}$. As discussed earlier in Section 3.2, this can be computationally expensive if a single NN has to process $Z_{i}$ entirely. For instance, if 50 Doppler bins are considered (i.e., 200 Hz bins, such that the DNN has more resolution to identify the CAF peak) in generating the CAF for a GPS L1 C/A signal, those images would be 4000×50-dimensional for the $f_{s}$ considered in this work. Alternatively, if $Z_{i}$ is split into smaller images of size 11×36 (read as: Doppler × delay), there are a total of 158,600 low-dimensional subimages to be efficiently processed by the NN, potentially in parallel. A sub-image with the size 11×36 was considered to provide a reasonable tradeoff between sub-image size and model complexity; since, in this method, we consider the subimages with the complete CAF peak exactly in the middle of the subimage. Considering that subimages of smaller sizes than the current size might cause issues, in which the CAF peak might not be included in any of the subimages, larger subimage sizes would cause multiple peaks to appear and higher computational complexity.

An interpretation of the sliding concept proposed in this article has some similarities to how the convolutional layers in a CNN are processed through the so-called *stride* parameter. In the proposed scheme, the CAF is scanned in smaller windows, each of which can contain the signal peak of interest. This peak, in contrast to peaks generated by random noise, shows a correlation in the delay and (more noticeably) in the Doppler domains that can be exploited by the NN classifier. The NN-based classifier uses a subimage (so a collection of delay/Doppler bins) to produce a classification result, as opposed to classical GNSS acquisition methods which use bin-by-bin detection strategies (i.e., every delay/Doppler bin is compared with a threshold to decide for presence/absence of a signal), which seems to bring accuracy benefits to the NN.

In order to train the NN-based classifier, the generated dataset contained either signal-plus-noise ($H_{1}$) or noise only ($H_{0}$) snapshots, which were then split into subimages, as shown in Figure 4. Since there will be many subimages that contain the CAF peak, in this method, the specific subimage that contains the complete CAF peak exactly in the middle of the subimage is considered as a correctly detected peak, and these types of snapshots are fed to the NN for training. The classifier learned its parameters by observing a set of 3000 input/output pairs in a supervised manner. The output of the NN was a *softmax* layer with dropout, such that the resulting outcome of the NN are the binary class probabilities required to compute the test in (19), or its noncoherent version in (23).

The particular convolutional neural network’s structure was based on the architecture in [52], containing 7 convolution layers and 3 fully connected layers. Each convolutional layer was followed by a batch normalization layer and a ReLu activation function. The batch normalization layers are used to normalize the activation and gradients propagation through the network between the convolution and ReLu layers, which is known to speed up network training tasks [55]. Each fully connected layer follows up with the ReLu activation function and a dropout layer with 1/2 probability rate. Since the task is a binary classification, the last fully connected layer contains two neurons, predicting the posterior probabilities of each hypothesis. Other relevant training options were specified, such as the use of a stochastic gradient descent with momentum (SGDM) optimizer with an initial learning rate of 0.001. The maximum number of epochs, which is a full training cycle on the entire training dataset, was set to 30 and, at every epoch, the data were shuffled. After 20 epochs, the learning rate dropped by a factor of 0.1.

The loss, which SGDM optimizes, was the cross-entropy loss, and the accuracy was defined as the percentage of inputs that the network classified correctly. Particularly, the validation accuracy after training the model reached 93%, which is deemed a high enough rate to consider the NN ready for deployment. Section 5 provides testing results of the trained and validated model, showing ROC performances and other relevant metrics.

## 5. Results

The proposed data-driven signal detection scheme was tested, and its performance was assessed through simulated data. The details of the model can be consulted in Section 3, while the training process is discussed in Section 4. While the training of the model was conducted using CAF images produced by 1 ms coherent integration times, the overall method was tested with and without noncoherent integration schemes. Particularly, when considering noncoherent integration times, K=6 was considered.

**Figure 6:**
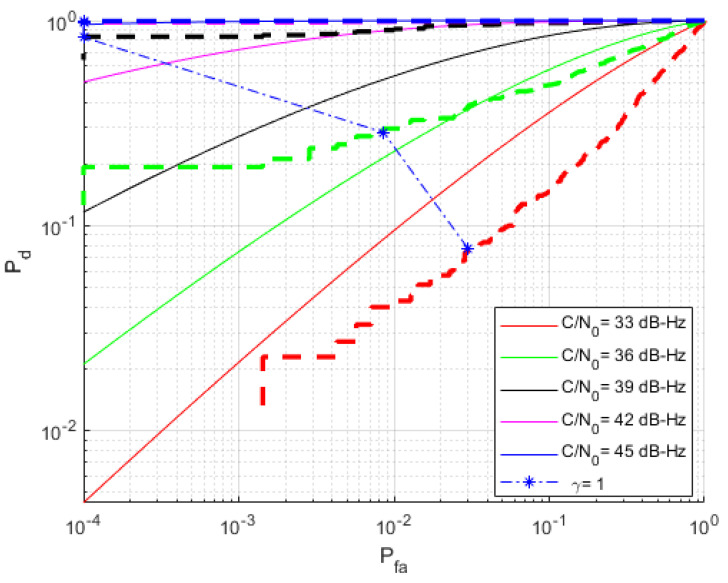
ROC curves (detection versus false-alarm probability) for a recording with 1 ms coherent integration and K=6 noncoherently processed blocks. Several relevant $C/N_{0}$ values are shown. The detection performance of the proposed scheme (dashed lines) is compared with the theoretical performance of standard methods (solid lines).

To assess the performance of the detection scheme, its ROC curves were empirically obtained through simulations and compared with the theoretical performance of standard methods (as reviewed in Section 2.2 or more in-depth in [10]). Figure 6 provides results for K=6 noncoherent integration periods (dashed lines), as compared with the theoretical performance (solid lines) of standard methods (aiming at maximizing the CAF) with the coherent/noncoherent values. Results show that whereas at low $C/N_{0}$ values the proposed method can barely achieve the state-of-the-art performance, it does remarkably well at larger $C/N_{0}$ values. It is worth noting that the improved performance starts at $C/N_{0}$ as low as 36 dB-Hz, which could be considered to be on the limit of the moderate–low range.

An explanation is that for low $C/N_{0}$, the DNN cannot extract the relevant features from the corresponding *subimage*, $Z_{i}^{\left(m,n\right)}$, but at higher $C/N_{0}$ values, the relevant features can be extracted, and the classification task successfully performed with desirable $P_{d}$ and $P_{fa}$ rates.

Surprisingly, the results in Figure 6 also show that the proposed data-driven scheme outperforms current performance bounds, suggesting it is leveraging additional information. This additional information comes from the prior that is embedded in the classifier through the seen training dataset. More precisely, whereas standard methods are based on the maximization of the CAF and identifying the associated bin, the proposed data-driven method exploits the correlation across neighboring bins to compute the class probability. That is, the classifier uses a subimage that contain a detail of the CAF that, under $H_{1}$, contains the relevant waveform of the CAF and its delay/Doppler correlated values.

On the other side, it is worth mentioning that the performance of the scheme for K=1 is substantially degraded compared with standard model-based schemes. This is explained by the low signal-to-noise ratio in this situation, as argued similarly earlier.

A benefit of the Bayes test approach considered in this work is that the adjustment of the detection threshold γ in (19) (or the one in (23) when K>1) has a probabilistic interpretation: how much larger the posterior probability of $H_{1}$ has to be from $H_{0}$ to be accepted. A reasonable choice would be γ=1, such that one picks the class with the largest posterior probability. Figure 6 shows the ROC results when such a choice is made for the detection threshold.

**Figure 7:**
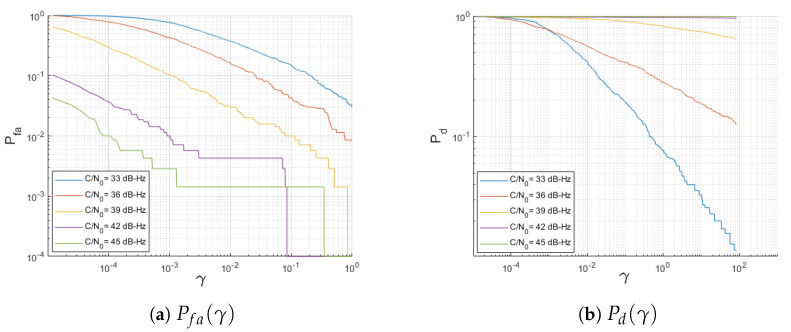
Probabilities for a 1 ms coherently integrated snapshot, K=6 noncoherent processing, and a variety of $C/N_{0}$ values.

According to the results, γ=1 provides good results for $C/N_{0}>36$ dB-Hz, with low false alarm and outstanding detection probabilities. For the sake of completeness, Figure 7 shows the false alarm and the detection probabilities corresponding to the ROC in Figure 6.

**Figure 8:**
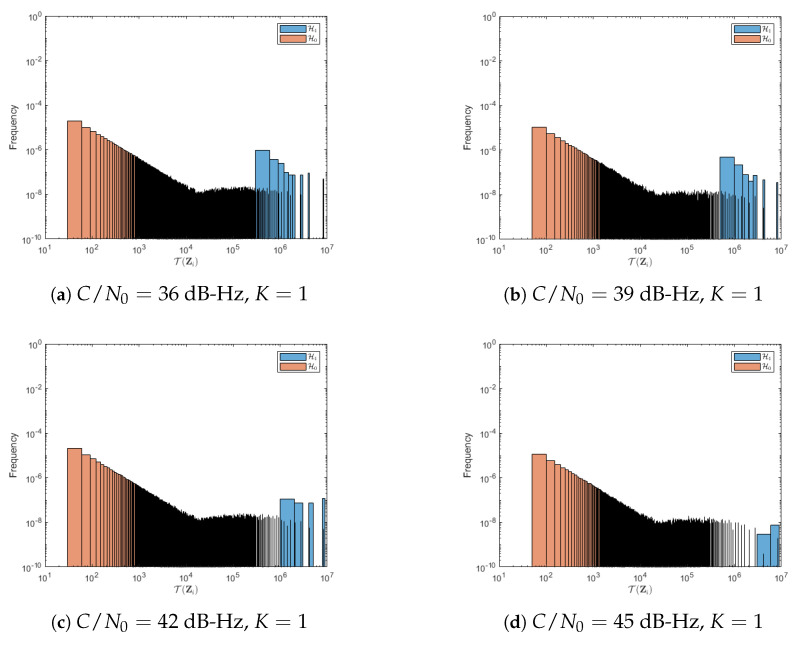
Test statistic histograms under $H_{0}$ and $H_{1}$ hypotheses for a 1 ms coherent integration time for a range of relevant $C/N_{0}$ values. The two histograms have overlapping areas, which suggests poor detection performance in these conditions.

**Figure 9:**
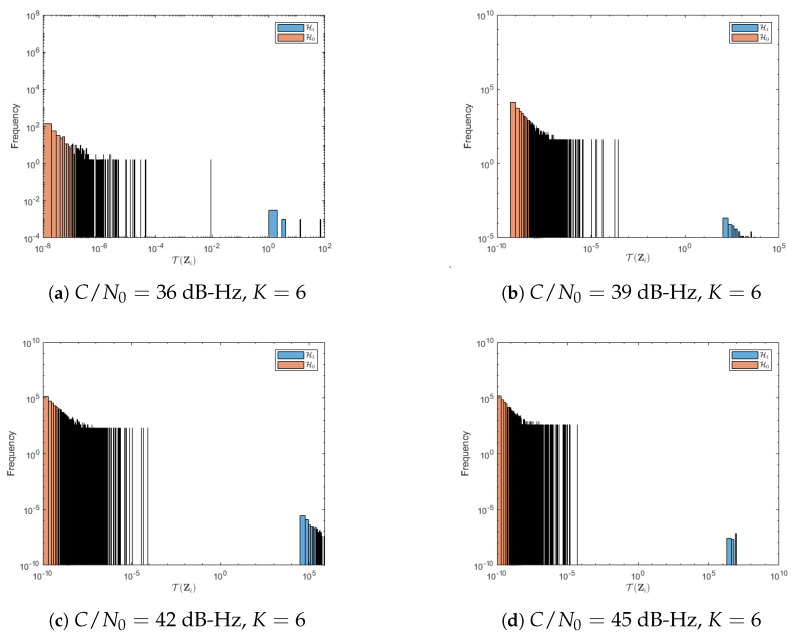
Test statistic histograms under $H_{0}$ and $H_{1}$ hypotheses for a 1 ms coherent integration time and K=6 noncoherent integrations for a range of relevant $C/N_{0}$ values. In this case, the two histograms are clearly separated, which supports the performance results in Figure 6.

The impact of low $C/N_{0}$ values on ROC curves is further explained by the histograms of the test statistic $T(Z_{i}^{\left(m,n\right)})$, which are shown in Figure 8 and Figure 9 for the cases of K=1 and K=6, respectively. Recall that one would like to have the histograms under $H_{0}$ and $H_{1}$ as *distant* as possible, which happens for large $C/N_{0}$ but clearly does not for low $C/N_{0}$ values. In Figure 9, the empirical distributions can be clearly distinguished in comparison with Figure 8, however, a sample from $T(Z_{i})$ cannot be statistically discerned between both distributions.

More precisely, without the noncoherent fusion rule, it is hard for the DNN to distinguish the difference between noise and signal subimages, particularly when the $C/N_{0}$ is low, which is shown in Figure 8. Although for large $C/N_{0}$ the separation increases, it is still far from desirable ROC regions. On the other hand, when noncoherent integration is considered, Figure 9 shows that there is no overlap between the two histograms, which causes an increased detection accuracy. In summary, when the signal power is high enough (or when noncoherent integration is used to increase that power), the DNN classifier performs remarkably well, even exceeding current state-of-the-art well-known performance results.

## 6. Conclusions and Future Work

Deep learning is a powerful data-driven tool which is increasingly being used in multiple fields and applications. This work proposes to use deep learning as a substitute to standard GNSS signal acquisition processing, a well-understood block present in all GNSS receivers. The proposed approach leverages a DNN classifier to output posterior class probabilities when the input is a region of the CAF for a specific satellite. The splitting of the CAF enables the flexible use of the method on CAFs of different dimensions (depending on the delay/Doppler bin sizes), as well as allowing for the parallelization of the process through multiple smaller DNN models. It is shown that the deep learning method can outperform standard approaches, even exceeding their fundamental limits in moderate-to-high signal-to-noise ratios. This result can be explained by the fact that standard methods are based on the bin maximization of the CAF, whereas the proposed data-driven method exploits the correlation across neighboring bins. Additionally, an optimal fusion rule is provided in order to extend the methodology to noncoherent integration schemes, which is also seen to improve the overall classification performance. The use of deep learning for advanced GNSS receiver design is in its infancy, from which many research directions can be foreseen. In the context of the framework proposed in this paper, future work includes the study of the proposed deep learning methodology in the presence of other sources of errors, such as receiver clock instabilities, higher receiver dynamics and the presence of jamming interferences or spoofing signals, as well as testing on real datasets.

## References

[1] Dardari D., Falletti E., Luise M. (2011). Satellite and Terrestrial Radio Positioning Techniques: A Signal Processing Perspective.

[2] Morton Y.J., van Diggelen F., Spilker J.J., Parkinson B.W., Lo S., Gao G. (2021). Position, Navigation, and Timing Technologies in the 21st Century: Integrated Satellite Navigation, Sensor Systems, and Civil Applications.

[3] Dardari D., Closas P., Djurić P.M. (2015). Indoor tracking: Theory, methods, and technologies. IEEE Trans. Veh. Technol..

[4] Williams N., Darian P.B., Wu G., Closas P., Barth M. (2022). Impact of Positioning Uncertainty on Connected and Automated Vehicle Applications. SAE Int. J. Connect. Autom. Veh..

[5] Amin M.G., Closas P., Broumandan A., Volakis J.L. (2016). Vulnerabilities, threats, and authentication in satellite-based navigation systems [scanning the issue]. Proc. IEEE.

[6] Kassas Z.M., Closas P., Gross J. (2019). Navigation systems panel report navigation systems for autonomous and semi-autonomous vehicles: Current trends and future challenges. IEEE Aerosp. Electron. Syst. Mag..

[7] Kaplan E., Hegarty C. (2005). Understanding GPS: Principles and Applications.

[8] Misra P., Enge P. (2006). Global Positioning System: Signals, Measurements and Performance.

[9] Tsui J.B. (2005). Fundamentals of Global Positioning System Receivers: A Software Approach.

[10] Borio D. (2008). A Statistical Theory for GNSS Signal Acquisition. Ph.D. Thesis.

[11] Mathis H., Flammant P., Thiel A. (2003). An analytic way to optimize the detector of a post-correlation FFT acquisition algorithm. Quadrature.

[12] Whalen A. (2013). Detection of Signals in Noise.

[13] Lehner A., Steingass A. A novel channel model for land mobile satellite navigation. Proceedings of the Institute of Navigation Conference ION GNSS.

[14] Borio D., Closas P. (2017). A fresh look at GNSS anti-jamming. Inside GNSS.

[15] Borio D. Robust signal processing for GNSS. Proceedings of the 2017 European Navigation Conference (ENC).

[16] Borio D. (2017). Myriad Non-Linearity for GNSS Robust Signal Processing. IET Radar Sonar Navig..

[17] Borio D., Closas P. (2018). Complex Signum Non-Linearity for Robust GNSS Signal Mitigation. IET Radar Sonar Navig..

[18] Borio D., Li H., Closas P. (2018). Huber’s Non-Linearity for GNSS Interference Mitigation. Sensors.

[19] Borio D., Closas P. (2019). Robust Transform Domain Signal Processing for GNSS. Navigation.

[20] Li H., Borio D., Closas P. Dual-Domain Robust GNSS Interference Mitigation. Proceedings of the International Technical Meeting of The Satellite Division of the Institute of Navigation (ION GNSS+ 2019).

[21] Borio D., Gioia C. (2021). GNSS interference mitigation: A measurement and position domain assessment. Navig. J. Inst. Navig..

[22] Borhani-Darian P., Closas P. Deep Neural Network Approach to GNSS Signal Acquisition. Proceedings of the 2020 IEEE/ION Position, Location and Navigation Symposium (PLANS).

[23] Azari B., Cheng H., Soltani N., Li H., Li Y., Belgiovine M., Imbiriba T., D’Oro S., Melodia T., Wang Y. (2022). Automated deep learning-based wide-band receiver. Comput. Netw..

[24] Dampf J., Pany T., Bär W., Winkel J., Stöber C., Fürlinger K., Closas P., Garcia-Molina J. (2015). More than we ever dreamed possible: Processor technology for GNSS software receivers in the year 2015. Inside GNSS.

[25] Siemuri A., Kuusniemi H., Elmusrati M., Välisuo P., Shamsuzzoha A. Machine Learning Utilization in GNSS—Use Cases, Challenges and Future Applications. Proceedings of the 2021 International Conference on Localization and GNSS (ICL-GNSS).

[26] Abdallah A.A., Kassas Z.M. Deep learning-aided spatial discrimination for multipath mitigation. Proceedings of the 2020 IEEE/ION Position, Location and Navigation Symposium (PLANS).

[27] Zhang G., Xu P., Xu H., Hsu L.T. (2021). Prediction on the Urban GNSS Measurement Uncertainty Based on Deep Learning Networks With Long Short-Term Memory. IEEE Sens. J..

[28] Huang P., Rizos C., Roberts C. (2018). Satellite selection with an end-to-end deep learning network. GPS Solut..

[29] Li H., Borhani-Darian P., Wu P., Closas P. Deep Learning of GNSS Signal Correlation. Proceedings of the 33rd International Technical Meeting of the Satellite Division of The Institute of Navigation (ION GNSS+ 2020).

[30] Savas C., Dovis F. Multipath Detection based on K-means Clustering. Proceedings of the 32nd International Technical Meeting of the Satellite Division of The Institute of Navigation (ION GNSS+ 2019).

[31] Suzuki T., Kusama K., Amano Y. NLOS Multipath Detection using Convolutional Neural Network. Proceedings of the 33rd International Technical Meeting of the Satellite Division of the Institute of Navigation (ION GNSS+ 2020).

[32] Munin E., Blais A., Couellan N. Convolutional neural network for multipath detection in GNSS receivers. Proceedings of the 2020 International Conference on Artificial Intelligence and Data Analytics for Air Transportation (AIDA-AT).

[33] Caparra G., Zoccarato P., Melman F. Machine Learning Correction for Improved PVT Accuracy. Proceedings of the 34th International Technical Meeting of the Satellite Division of The Institute of Navigation (ION GNSS+ 2021).

[34] Manesh M.R., Kenney J., Hu W.C., Devabhaktuni V.K., Kaabouch N. Detection of GPS spoofing attacks on unmanned aerial systems. Proceedings of the 2019 16th IEEE Annual Consumer Communications & Networking Conference (CCNC).

[35] Borhani-Darian P., Li H., Wu P., Closas P. Deep Neural Network Approach to Detect GNSS Spoofing Attacks. Proceedings of the 33rd International Technical Meeting of the Satellite Division of the Institute of Navigation (ION GNSS+ 2020).

[36] Tohidi S., Mosavi M.R. Effective detection of GNSS spoofing attack Using A multi-layer perceptron neural network classifier trained by PSO. Proceedings of the 2020 25th International Computer Conference, Computer Society of Iran (CSICC).

[37] Calvo-Palomino R., Bhattacharya A., Bovet G., Giustiniano D. Short: LSTM-based GNSS Spoofing Detection Using Low-cost Spectrum Sensors. Proceedings of the 2020 IEEE 21st International Symposium on “A World of Wireless, Mobile and Multimedia Networks” (WoWMoM).

[38] Semanjski S., Muls A., Semanjski I., De Wilde W. Use and validation of supervised machine learning approach for detection of GNSS signal spoofing. Proceedings of the 2019 International Conference on Localization and GNSS (ICL-GNSS).

[39] Morales Ferre R., de la Fuente A., Lohan E.S. (2019). Jammer classification in GNSS bands via machine learning algorithms. Sensors.

[40] Louis A., Raimondi M. Neural Network based Evil WaveForms Detection. Proceedings of the 33rd International Technical Meeting of the Satellite Division of The Institute of Navigation (ION GNSS+ 2020).

[41] Brum D., Veronez M.R., de Souza E.M., Koch I.É., Gonzaga L., Klein I., Matsuoka M.T., Rofatto V.F., Junior A.M., dos Reis Racolte G.E. A Proposed Earthquake Warning System Based on Ionospheric Anomalies Derived From GNSS Measurements and Artificial Neural Networks. Proceedings of the IGARSS 2019—2019 IEEE International Geoscience and Remote Sensing Symposium.

[42] Alshaye M., Alawwad F., Elshafiey I. Hurricane tracking using Multi-GNSS-R and deep learning. Proceedings of the 2020 3rd International Conference on Computer Applications & Information Security (ICCAIS).

[43] Yan Q., Huang W. (2018). Sea ice sensing from GNSS-R data using convolutional neural networks. IEEE Geosci. Remote Sens. Lett..

[44] Linty N., Farasin A., Favenza A., Dovis F. (2018). Detection of GNSS ionospheric scintillations based on machine learning decision tree. IEEE Trans. Aerosp. Electron. Syst..

[45] Liu Y., Morton Y., Jiao Y. Application of Machine Learning to Characterization of GPS L1 Ionospheric Amplitude Scintillation. Proceedings of the 2018 IEEE/ION Position, Location and Navigation Symposium (PLANS).

[46] Selbesoglu M.O. (2020). Prediction of tropospheric wet delay by an artificial neural network model based on meteorological and GNSS data. Eng. Sci. Technol. Int. J..

[47] Vilà-Valls J., Linty N., Closas P., Dovis F., Curran J.T. (2020). Survey on signal processing for GNSS under ionospheric scintillation: Detection, monitoring, and mitigation. Navig. J. Inst. Navig..

[48] Imbiriba T., Demirkaya A., Duník J., Straka O., Erdoğmuş D., Closas P. Hybrid Neural Network Augmented Physics-based Models for Nonlinear Filtering. Proceedings of the FUSION Conference.

[49] O’Shea T., Roy T., Clancy T. (2018). Over-the-air deep learning based radio signal classification. IEEE J. Sel. Top. Signal Process..

[50] Yan Q., Huang W., Moloney C. (2017). Neural networks based sea ice detection and concentration retrieval from GNSS-R delay-Doppler maps. IEEE J. Sel. Top. Appl. Earth Obs. Remote Sens..

[51] White C., Neiswanger W., Savani Y. BANANAS: Bayesian Optimization with Neural Architectures for Neural Architecture Search. Proceedings of the AAAI Conference on Artificial Intelligence.

[52] Liu S., Deng W. Very deep convolutional neural network based image classification using small training sample size. Proceedings of the 2015 3rd IAPR Asian Conference on Pattern Recognition (ACPR).

[53] Simonyan K., Zisserman A. (2014). Very deep convolutional networks for large-scale image recognition. arXiv.

[54] Pastor F., García-González J., Gandarias J.M., Medina D., Closas P., García-Cerezo A.J., Gómez-de Gabriel J.M. (2020). Bayesian and Neural Inference on LSTM-Based Object Recognition from Tactile and Kinesthetic Information. IEEE Robot. Autom. Lett..

[55] Mathworks Create Simple Deep Learning Network for Classification. https://www.mathworks.com/help/deeplearning/examples/create-simple-deep-learning-network-for-classification.html.

